# Readiness to Change Over Time: Change Commitment and Change Efficacy in a Workplace Health-Promotion Trial

**DOI:** 10.3389/fpubh.2018.00110

**Published:** 2018-04-23

**Authors:** Christian D. Helfrich, Marlana J. Kohn, Austin Stapleton, Claire L. Allen, Kristen Elizabeth Hammerback, K. C. Gary Chan, Amanda T. Parrish, Daron E. Ryan, Bryan J. Weiner, Jeffrey R. Harris, Peggy A. Hannon

**Affiliations:** ^1^Seattle-Denver Center of Innovation for Veteran-Centered and Value-Driven Care, US Department of Veterans Affairs, Seattle, WA, United States; ^2^Department of Health Services, School of Public Health, University of Washington, Seattle, WA, United States; ^3^Health Promotion Research Center, A CDC Prevention Research Center, Department of Health Services, University of Washington, Seattle, WA, United States

**Keywords:** readiness to change, implementation, change commitment, change efficacy, psychometric validation, workplace health promotion

## Abstract

**Introduction:**

Organizational readiness to change may be a key determinant of implementation success and a mediator of the effectiveness of implementation interventions. If organizational readiness can be reliably and validly assessed at the outset of a change initiative, it could be used to assess the effectiveness of implementation-support activities by measuring changes in readiness factors over time.

**Methods:**

We analyzed two waves of readiness-to-change survey data collected as part of a three-arm, randomized controlled trial to implement evidence-based health promotion practices in small worksites in low-wage industries. We measured five readiness factors: context (favorable broader conditions); change valence (valuing health promotion); information assessment (demands and resources to implement health promotion); change commitment (an intention to implement health promotion); and change efficacy (a belief in shared ability to implement health promotion). We expected commitment and efficacy to increase at intervention sites along with their self-reported effort to implement health promotion practices, termed wellness-program effort. We compared means between baseline and 15 months, and between intervention and control sites. We used linear regression to test whether intervention and control sites differed in their change-readiness scores over time.

**Results:**

Only context and change commitment met reliability thresholds. Change commitment declined significantly for both control (−0.39) and interventions sites (−0.29) from baseline to 15 months, while context did not change for either. Only wellness program effort at 15 months, but not at baseline, differed significantly between control and intervention sites (1.20 controls, 2.02 intervention). Regression analyses resulted in two significant differences between intervention and control sites in changes from baseline to 15 months: (1) intervention sites exhibited significantly smaller change in context scores relative to control sites over time and (2) intervention sites exhibited significantly higher changes in wellness program effort relative to control sites.

**Discussion:**

Contrary to our hypothesis, change commitment declined significantly at both Healthlinks and control sites, even as wellness-program effort increased significantly at HealthLinks sites. Regression to the mean may explain the decline in change commitment. Future research needs to assess whether baseline commitment is an independent predictor of wellness-program effort or an effect modifier of the HealthLinks intervention.

## Introduction

Organizational readiness to change is the psychological and behavioral preparedness of organizational members tasked with implementation of a new practice, policy, or technology (1). Organizational readiness is thought to be a key determinant of implementation success and a mediator of the effectiveness of implementation interventions (1–3). Readiness is a core construct in several dissemination and implementation frameworks (4–6).

If organizational readiness can be reliably and validly assessed at the outset of a change initiative, measures of readiness could be used prognostically to gain an accurate prediction of the likelihood of change success and diagnostically to identify specific weaknesses or deficits in readiness. If accurately measured, organizational readiness could be used in workplace health promotion efforts to target worksites for dissemination; to diagnose and address worksite-specific deficits in readiness; and to assess the effectiveness of implementation-support activities by measuring changes in readiness factors over time. Accurate organizational readiness could also be considered or intervened upon with implementation-support activities, such as information, training, and marketing materials.

We tested a previously developed survey designed specifically for assessing organizational readiness to implement evidence-based workplace health promotion practices (7). Our objective was to determine if the instrument was sensitive to changes in readiness factors over time and differences in readiness among workplaces participating in a randomized, controlled implementation trial receiving different implementation-support interventions. Our hope was that the readiness measure could ultimately be used in broader dissemination and implementation efforts to identify workplace-specific implementation barriers that can be addressed with implementation-support activities and potentially repeated to determine if support activities have been successful. However, this is only possible if the readiness measure is sensitive to changes in readiness factors over time and sensitive to improvements in readiness resulting from implementation-support activities. The purpose of this paper is to test the readiness measure’s sensitivity to changeover time in worksites that attempted to implement new health promotion practices.

## Materials and Methods

### Design

We analyzed two waves of survey data collected as part of a three-arm, randomized, controlled trial testing the effectiveness of HealthLinks, a workplace-health-promotion program (8). HealthLinks was developed in collaboration with the American Cancer Society and the University of Washington. It is tailored to the needs and capacities of small worksites to help them implement evidence-based practices for workplace health promotion. Worksites participating in HealthLinks receive an assessment of their current implementation of evidence-based practices; a tailored recommendations report; toolkits to support implementation of each of the practices; and onsite, telephone, and email assistance from a trained interventionist. As part of the trial, we developed and validated a readiness-to-change survey, with the goal of creating a survey that could be used in subsequent dissemination efforts (7).

HealthLinks aimed to increase the adoption and implementation of 11 evidence-based health promotion practices through provision of materials and onsite implementation assistance. The evidence-based health promotion practices, recommended by CDC’s Community Guide to Preventive Services (9) as compatible with worksites, focused on healthy eating, physical activity, tobacco cessation, and screening for breast, cervical, and colon cancers (Table 1).

**Table 1:** Evidence-based health promotion practices to be implemented as part of HealthLinks.

Behavior	Interventions Promoted in HealthLinks and HealthLinks+
Breast, cervical, and colon cancer screening	Distribute brochures and post posters to educate workers about cancer screening guidelines Provide brief education sessions at the worksite, including benefits of screening and information about costs/insurance coverage Promote the Washington Breast, Cervical, and Colon Health Program to uninsured workers; include information about local providers, screening free of charge, and treatment coverage for those diagnosed with cancer Promote benefits coverage at those worksites with insurance benefits

Healthy eating	For worksites that sell food, create policies to offer healthy options, label them, and price them competitively For all worksites, create policies to support offering healthy foods at meetings and events

Physical activity	Negotiate discounts at local gyms for workers Post “Use the Stairs” signs Offer ACS Active for Life program, an evidence-based program that offers individual choice of activity and builds social support

Tobacco cessation	Promote the Washington State Tobacco Quit Line *via* brochures and other small media; include information about quit line services Promote benefits coverage at those worksites with insurance coverage for tobacco cessation

HealthLinks enrolled small worksites in six low-wage industries in King County in Washington State. One intervention arm received only the HealthLinks program (Standard HealthLinks), one arm received HealthLinks plus support to form wellness committees (HealthLinks + Wellness Committee), and the third arm was a delayed control group. As part of the study, worksites completed surveys at baseline and 15 months to assess readiness factors and specific implementation efforts to implement health promotion practices (described below). The study protocol and baseline outcomes have been previously published (8).

### Conceptual Model

**Figure 1:**
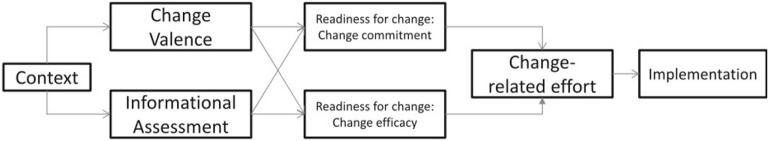
Theory of organizational readiness to change.

The readiness measures and analysis were guided by Weiner’s theory of organizational readiness to change (Figure 1) (1). It hypothesizes that organizational readiness to change comprises two collective, affective states: *change commitment* and *change efficacy*. Change commitment refers to an intention to implement a change that is shared across members of an organization. Change efficacy is defined as organizational members’ shared beliefs in their joint ability to engage in those courses of action necessary to implement a change. *Change-related effort*, which we hereafter refer to as *wellness-program effort*, is the collective effort of organizational members to execute a change, and is a function of both change commitment and change efficacy. While beyond the scope of the current analysis, wellness-program effort is expected to predict the actual extent of implementation of workplace wellness programs.

Change commitment and change efficacy are functions of *change valence* and *informational assessment*. Change valence is the extent to which members of an organization value the change. Reasons for why the change is valued can vary, and this construct does not assume that all members value it for the same reason, only that there exists a collective belief that the change is significant to the goals of the organization. Informational assessment refers to organizational members’ perceptions that the resources available to implement the change (human, financial, material, and informational) are sufficient to the demand.

Change valence and informational assessment are influenced in turn by *context*, which refers to the broader conditions that affect readiness to change, such as organizational culture, climate, resources, structure, and past experiences with implementing change. Relative to the other constructs in the model, context is not innovation-specific and should be more stable over time.

### Setting and Sample

HealthLinks was tested among small workplaces, defined as 20–200 employees, in low-wage industries in King County in Washington State. We selected industries by North American Industry Classification System (NAICS) codes: accommodation and food services; arts, entertainment, and recreation; education; health care and social assistance; retail trade; and other services excluding public administration. We required eligible worksites to have a minimum of 20% of their employees report to a physical site at least once per week; to have been in business for at least 3 years; and worksites could not already have a wellness committee in place. A total of 78 sites were enrolled, with 28 assigned to the HealthLinks arm; 26 assigned to the HealthLinks plus wellness committee arm; and 24 assigned to the delayed controls.

### Data Collection and Measures

This analysis used three measures: company characteristics, organizational readiness scales, and implementation-related efforts, referred to as *wellness program effort*. Company characteristics included type of industry, number of employees (size), for-profit vs. not-for-profit, proportion of full-time employees, and whether health insurance was offered to employees.

The readiness to change and wellness program-effort scales were previously developed for this study through a multi-stage validation process. First, we identified existing readiness scales that measured constructs in the Weiner readiness model, starting with the organizational readiness to change measure developed by Shea and colleagues (10) based on the Weiner model, as well other readiness to change surveys (11, 12), and a prior wellness-program survey (13). We then conducted think aloud interviews with employers similar to our study sample to evaluate and revise items for comprehension and appropriateness. Finally, we piloted the survey with a sample of 201 small Washington employers in the same industries as HealthLinks (separate from our HealthLinks sample) in order to assess scale reliability and criterion validity. The latter included a path analysis of scales to confirm that associations among the scales conformed to Weiner’s theory of organizational readiness. Survey development and validation procedures and findings were reported in detail in a prior paper (7).

Readiness items (Table S1 in Supplementary Material) were scored on 5-point Likert scales (1 = strongly disagree, 5 = strongly agree). The context scale comprised 10 items assessing leadership, management, and opinion leaders’ willingness to trying new things; whether they reward creativity and innovation; whether they promote teambuilding to solve worksite problems; and whether they seek to improve workplace climate. The information assessment scale comprised five items assessing availability of staff time, financial resources, and employee and leadership champions for wellness programs. The change valence scale comprised four items assessing whether wellness programs would benefit the organization in terms of improving employee health, improving employee recruitment and retention, and reducing employee health-care costs. The change commitment scale comprised five items assessing senior leader, opinion leader, and collective commitment and motivation to start or improve a wellness program. The change efficacy scale comprised four items assessing collective skills, expertise, ability to manage workplace politics, and ability to obtain employee participation, while implementing a wellness program.

Wellness program effort was measured *via* five questions about implementation activities for wellness programs, such as having written wellness goals, a wellness committee and coordinator, and/or a health promotion or wellness budget. The fifth item, how much time the respondent thought s/he could spend on a wellness program, was not included in the original development of the wellness program effort scale. We added it here because time spent on wellness activities is an additional and concrete indicator of wellness program effort. The time spent on wellness program effort item was a five-point (1–5) Likert-type scale. Yes–no items, initially coded in the data as yes = 1, no = 0, were re-coded yes = 5, no = 1 to align with the scoring of scale items throughout the readiness survey instrument.

Data were collected through surveys conducted in person at baseline and *via* telephone at 15-month follow-up. With the exception of a section on satisfaction with the HealthLinks program at follow-up, worksites answered identical sets of questions at baseline and follow-up. Surveys were completed by the primary worksite contact for the study, usually, the Human Resource manager, who would be involved in any workplace health promotion efforts. Delayed control sites received the HealthLinks intervention after data collection ended.

### Analysis

We examined the means of measures at baseline and 15 months, and among intervention groups, and tested mean differences using a paired *t*-test. We used a significance level of *p* ≤ 0.05. We then examined the association between readiness and wellness-program effort score change and intervention groups using linear regression models, adjusting for worksite size (20–49 vs. 50–200), and industry (arts, entertainment, and recreation/education/health care and social assistance v. accommodation and food services/other services excluding public administration/retail trade), which were the blocking variables for trial randomization and have previously been found to be related to workplace health promotion practices (13). Our hypothesis was that change commitment, change efficacy, and wellness-program effort would increase significantly from baseline to 15 months at intervention sites while not changing significantly at control sites. We used the difference scores of the baseline and 15-month surveys as our outcome measures, and there was a single survey respondent per site.

Our initial analyses examined each of the three study arm sites compared to the other two study arm sites, and the two intervention arm sites (HealthLinks and HealthLinks + Wellness Committee) compared to control sites. As we saw few differences between the intervention sites, we focus the results below on the analyses comparing the combined intervention sites to control sites.

Analyses were conducted with STATA version 15 (College Station, TX, USA).

### Human Subjects Approval

The University of Washington Institutional Review Board approved all study materials and procedures. This study is registered at https://Clinicaltrials.gov: NCT02005497.

## Results

All 78 worksites completed baseline surveys; 72 (92.3%) completed follow-up surveys. Our analyses included the 72 worksites with complete baseline and follow-up data. Intervention and control sites did not differ in industry characteristics (Table 2).

**Table 2:** Characteristics of participating companies by study arm.

Intervention arm *N* = 72							

	Control (*n* = 21)		Standard HealthLinks (*n* = 26)		HealthLinks with Wellness Committee (*n* = 25)		
				
Company characteristics	Mean (SD)	Percent	Mean (SD)	Percent	Mean (SD)	Percent	*p-*Value
Total employees	75.81 (47.0)	–	72.5 (44.4)		74.44 (52.4)		0.97
Annual salary	$37,031 (11,369)	–	%38,369 (12,445)		$42,540 (14,405)		0.41
Percent full-time employees	71.7 (25.0)		76.3 (22.5)		75.08 (26.4)		0.82
Percent union membership	0.0 (0.0)		6.5 (21.5)		2.48 (12.0)		0.34
Company tax status							0.86
Non-profit		61.9		53.9		56.0	
For profit		38.1		46.2		44.0	
Company offers health insurance to employees		90.5		100		96.0	0.28
Company is self-insured		0.0		3.9		4.2	
Employees eligible for health insurance		85.6		83.5		79.7	
Employees enrolled in health insurance		82.2		82.2		81.3	
Industry^a^							0.38
Accommodation and food services		14.3		7.7		8.0	
Arts, entertainment, and recreation		0.0		0.0		12.0	
Educational services		14.3		7.7		8.0	
Health care and social assistance		38.1		50.0		44.0	
Other services (except public administration)		33.3		11.5		16.0	
Retail trade		0.0		23.1		12.0	

*^a^Industry as identified by NAICS code*.

Three readiness scales failed to meet reliability thresholds, as measured by Cronbach’s alpha (Table 3): Change valence (Cronbach’s alpha = 0.66 at baseline, 0.67 at 15 months), information assessment (0.64 at baseline, 0.54 at 15 months), and change efficacy (0.52 at baseline, 0.63 at 15 months). Context (0.72 at baseline, 0.79 at 15 months) and change commitment (0.72 at baseline, 0.71 at follow-up) met reliability thresholds. We only report subsequent findings for scales that exhibited reliability. We did not calculate alpha statistics for wellness program effort because four of the five items were dichotomous, which are not suitable for Crohnbach’s alpha.

**Table 3:** Organizational readiness to change scale means and reliabilities and means of Wellness Program Effort.

		Baseline			15 months		
			
		Mean	SD	Alpha^a^	Mean	SD	Alpha^a^
Readiness scales	Context	3.57	0.47	***0.72***	3.48	0.54	***0.79***
	Change valence	4.00	0.47	0.66	3.90	0.54	0.67
	Information assessment	3.63	0.60	0.64	3.55	0.56	0.54
	Change commitment	3.66	0.56	***0.72***	3.35	0.58	***0.71***
	Change efficacy	3.48	0.58	0.52	3.39	0.59	0.63

Implementation effort	Wellness program effort	1.25	0.43	N/A	1.57	0.71	N/A

*^a^We used a cutoff of 0.70 for reliability; alpha coefficients that met or exceeded threshold are bold italic*.

When assessing the differences between baseline and 15-month scores (Table 4), change commitment declined significantly for both control (−0.39) and interventions sites (−0.29), while context did not change for either control or intervention sites. When examining the change from baseline to 15 months for each intervention arm separately, the sites in the HealthLinks + wellness committee arm did not see a significant difference in change commitment. Wellness program effort, the proximal outcome, increased significantly for intervention sites (0.73) but did not change for control sites.

**Table 4:** Change in organizational readiness to change factors between baseline and 15 months.

		Baseline		15 months		Difference^1^
				
		Mean	SD	Mean	SD	–
Context	Control	3.48	0.40	3.62	0.46	0.14
	Intervention^2^	3.61	0.49	3.43	0.57	−0.18

Change valence	Control	3.96	0.42	3.95	0.55	−0.01
	Intervention	4.01	0.49	3.87	0.54	−0.14
Information assessment	Control	3.73	0.57	3.59	0.53	−0.14
	Intervention	3.59	0.61	3.53	0.58	−0.06

Change commitment	Control	3.69	0.57	3.30	0.59	**−*0.39***
	Intervention	3.65	0.56	3.37	0.58	**−*0.29***

Change efficacy	Control	3.51	0.61	3.40	0.60	−0.11
	Intervention	3.46	0.57	3.39	0.59	−0.07

Wellness program effort	Control	1.15	0.32	1.20	0.43	0.05
	Intervention	1.29	0.47	2.02	0.98	***0.73***

*^1^Differences in bold italic are mean values from baseline to 15 months that are significant at p-value ≤ 0.05*.

*^2^Intervention combines the Standard HealthLinks and + Wellness Committee groups*.

When assessing the differences between intervention and control sites for each scale and the outcome at each time period (baseline and 15 months), the only significant difference was for wellness program effort at 15 months (1.20 controls, 2.02 intervention, *p* < 0.05) (Table 5).

**Table 5:** Differences between intervention and control sites in organizational readiness to change and wellness program effort for baseline and 15-month results.

		Control	Intervention	Difference^a^
Context	Baseline	3.48	3.61	0.13
	15 months	3.62	3.43	−0.19

Change valence	Baseline	3.96	4.01	0.05
	15 months	3.95	3.87	−0.08

Information assessment	Baseline	3.73	3.59	−0.14
	15 months	3.59	3.53	−0.06

Change commitment	Baseline	3.69	3.65	−0.03
	15 months	3.30	3.37	0.07

Change efficacy	Baseline	3.51	3.46	−0.05
	15 months	3.40	3.39	−0.02

Wellness program effort	Baseline	1.15	1.29	0.13
	15 months	1.20	2.02	***0.82***

*^a^Differences in mean values between intervention and control sites significant at p-value ≤ 0.05 are in bold italic*.

Regression analyses resulted in two significant differences between intervention and control sites in changes over the 15 months from baseline to follow-up (Table 6). First, the change in context scores from baseline to follow-up was significantly lower for intervention sites relative to control sites. Second, intervention sites exhibited significantly higher changes in wellness program effort relative to control sites. There were no differences between intervention and control sites in the change in change commitment from baseline to follow-up.

**Table 6:** Regression model results for differences between intervention and control sites in change from baseline to 15 months in context, change commitment and wellness program effort.

		Coefficient^a^	SE	*t*	*p* > |t|	95% conf. interval	
Change in context	Intervention	**−*0.31***	0.12	−2.59	0.01	−0.56	−0.07
	Size	0.01	0.11	0.11	0.91	−0.21	0.23
	Industry	−0.20	0.11	−1.77	0.08	−0.42	0.03
	Constant	0.24	0.13	1.87	0.07	−0.02	0.50

Change in change commitment	Intervention	0.11	0.17	0.63	0.53	−0.24	0.46
	Size	0.09	0.16	0.60	0.55	−0.22	0.41
	Industry	0.08	0.16	0.52	0.60	−0.24	0.40
	Constant	−0.49	0.19	−2.62	0.01	−0.86	−0.12

Change in wellness program effort	Intervention	***0.69***	0.21	3.32	0.00	0.28	1.10
	Size	0.08	0.19	0.44	0.67	−0.30	0.46
	Industry	0.38	0.19	2.00	0.05	0.00	0.77
	Constant	−0.22	0.22	−0.97	0.34	−0.66	0.23

*^a^Coefficients significant at p-value ≤ 0.05 are in bold italic*.

In secondary analyses, we evaluated the reliability of scales, following the procedures we used in our original validation study, to determine if scale reliability could be improved by eliminating items that had an item-rest correlation of 0.20 or lower. This procedure improved scale reliability but did not result in any change in the scales meeting our threshold of 0.70 (results available upon request).

## Discussion

Contrary to our hypothesis, change commitment declined significantly at Healthlinks sites, even as wellness-program effort increased significantly. One explanation for this apparent incongruity (declining commitment in the face of increasing effort) could be change fatigue: a gradual exhaustion of participants’ motivation over time as a consequence of their sustained change efforts. However, change commitment declined equally at control sites who were not engaged in any change efforts. The more likely explanation is regression to the mean. Sites were recruited over a period of 10 months and, most likely, motivation to engage in workplace health promotion varies randomly over time. Motivation to engage in workplace health promotion almost certainly correlated with interest in participating in the study, and sites that enrolled in the study were probably often randomly waxing in motivation at the time they decided to enroll. The decline in their 15-month scores may just represent a return to something closer to their average motivation or commitment to workplace wellness. We see indirect evidence of regression to the mean from comparing the change commitment scores observed in this study to the scores observed in the cross-sectional survey used in our prior scale-validation study (7): the mean scores on change commitment in that survey was 3.31, nearly identical to the change commitment scores at 15 months.

When analyzing changeover time, we also found that the difference in context scores from baseline to 15 months was significantly smaller at intervention sites relative to controls. The context scale measures attitudes and actions of senior leaders, managers, and opinion leaders related to workplace climate, creativity, innovation, and team-building to solve worksite problems. Through efforts to implement worksite health promotion practices, the HealthLinks intervention could have helped make deficiencies in those attitudes and actions more apparent. However, neither intervention nor control sites exhibited significant changes in context over time in bivariate analyses; it is only in comparing that changeover time that it is statistically different between intervention and control sites. The reason we use control sites is to identify and adjust for spurious associations unrelated to our intervention, such as secular trends. In this instance, the adjusted analysis using control sites is not isolating the effects of the intervention from secular effects, it is actually producing a new significant association for context that we do not observe otherwise. We think this is probably a random finding. Our primary conclusion is not that context, as we conceptualized and measured it, actually degraded as a result of HealthLinks, but rather that it had no material association.

Meanwhile, the scales measuring change efficacy, change valence, and informational assessment exhibited poor reliability and, consequently, we cannot draw any conclusions about the sensitivity of these measures to differences over time or among study arms. The poor reliability of these scales is perplexing; our prior validation of the survey, which included concurrent validation using a large sample of employers similar to the present study sample, found good reliability and criterion validity (7).

One explanation for the poor reliability may be a combination of sample size and systematic measurement error. Shevlin and colleagues have used Monte Carlo simulations to show that alpha coefficients are highly sensitive to the combination of sample size and the presence of measurement error, and the differences we found between our validation and trial data are generally within the differences they observed (14). We know our trial sample size was significantly lower (*n* = 72) than our validation study (*n* = 201). In addition, we might expect that the validation study (but not the trial) was susceptible to systematic error due to “halo effect,” because the validation study assessed readiness factors concurrently with extent of workplace health promotion practice. Halo effect is a type of inferential bias in which individuals form a general impression of someone or something and infer other qualities from that general impression, e.g., inferring an individual’s leadership qualities from how well one likes the individual (15). Cross-sectional criterion validation, in which we assess the criterion outcome at the same time as we assess readiness factors, is particularly susceptible to halo effect because the respondent already knows the outcome as they respond to questions about their readiness to achieve that outcome (15).

This article makes several contributions to the broader literature on change readiness. First, ours is the only study we are aware of to test the sensitivity of an organizational readiness-to-change measure to changes over time, and the findings ran contrary to our hypotheses. Our study used experimental manipulation that successfully induced greater implementation efforts among intervention sites, creating a scenario in which we had a strong theoretical rationale for expecting significantly greater change commitment and change efficacy over time at intervention sites relative to control sites. Yet, we observed no differences between intervention and control sites in commitment, and contrary to expectation, observed declining commitment over time among all sites. This is important because change commitment and change efficacy and related affective constructs such as intention and motivation, are central to most organizational readiness to change measures, and the vast majority of empirical work in this area has historically been cross-sectional or using other designs that are susceptible to bias, e.g., case studies, one-group pretest, posttest (16). We would like to see this experiment replicated in other health promotion contexts, and other implementation fields, to see if similar or different associations are found. That would help advance our underlying conceptual understanding of collective readiness as a prerequisite for effective organizational change.

Second, commitment is core to many implementation models as a mediator of implementation activities and implementation outcomes (1, 17, 18). Our findings raise the possibility that at least, in some settings, and for some changes, maintaining a high-level of change commitment may be immaterial for generating implementation effort. Our findings also raise questions about change efficacy, as we failed to find change efficacy associated with implementation effort. That may be due to issues with construct validity; unreliable measurement; sampling bias; or a combination. However, given our careful survey development and validation procedure (7) and the rigorous experimental design of these findings, at the very least, these results place a burden of proof on future studies in workplace health promotion that rely on change efficacy as a mechanism for change to demonstrate construct validity and measurement reliability.

Third and finally, many of our current implementation models and measures focus on attitudinal constructs, such as commitment, efficacy, and motivation, but this study suggests that more instrumental constructs, such as the planning and technical support that was provided by HealthLinks, may be more important variables in ensuring effective implementation. As noted, behavioral economics has repeatedly shown that people are often poor at predicting their own behaviors, or acting in ways that are consistent with their expressed goals and self-interest (19, 20). This experiment needs to be replicated, but if our findings are reproduced, one implication may be that measuring and influencing affective states is less useful than ensuring instrumental support, such as planning, which runs counter to some of the current thinking in the literature (21).

### Limitations

This study has several limitations that raise a variety of interesting questions. First, we found change commitment declined across study arms, likely due to regression to the mean. Our findings about change commitment also might reflect selection bias. The study population by definition only included volunteers, who were virtually certain to exhibit higher-than-average change commitment. This may have constrained the observed variation in change commitment. If we were able to randomize the whole population of small worksites in low-wage industries in King County to HealthLinks or control conditions, it is possible that we would observe significant changes in change commitment over time and significant differences between HealthLinks and control sites.

Second, baseline readiness factors notably change commitment and change efficacy might still be significant predictors of subsequent wellness program effort irrespective of the plasticity of the measures over time or their sensitivity to the effects of implementation strategies, such as HealthLinks. For example, baseline readiness factors including change commitment and change efficacy might be important independent predictors of subsequent wellness program effort. Or, they might be necessary but not sufficient conditions for successful implementation, and we could observe significant interactions between readiness factors and implementation strategy, such that sites with a high baseline-level of change commitment and change efficacy AND who receive the HealthLinks intervention would demonstrate much higher levels of wellness program effort than either sites with high baseline-level of change commitment and change efficacy OR receipt of the HealthLinks intervention alone. These questions were beyond the scope of the current analysis and are the focus of future work.

It is also possible that we need to rethink our conceptualization of readiness to change. At baseline, respondents were rating hypotheticals: how committed were they to engaging in a set of practices with which they generally did not have prior experience? How confident were they in their collective ability to implement health promotion practices? Research in cognitive psychology and behavioral economics has repeatedly shown people to be poor at predicting future behaviors, states, and feelings (19, 20). Participant ratings of their readiness to implement a new practice might be inherently unreliable until they have gained some experience with the practice. An alternative approach that could be tested in the future is to have participants estimate base rates: when they or others in their industry have attempted similar initiatives in the past, how often were they successful, and what were the main stumbling blocks and facilitators?

Our findings may have been biased by measurement error. The survey was fielded to a single individual, typically a human resources manager, identified by the employer as the contact for the study. Weiner’s theory (1) postulated that readiness is a shared construct, and ideally would be measured among all employees involved the change. It is possible that the individuals in our sample had incomplete or flawed insights into their companies’ readiness domains, and that a different sample, e.g., a broader sample of employees, or company executives, would produce more accurate measures of readiness and a different result. In more than a third of participating sites, there was turnover in the primary study contact completing these measures, and this could also introduce measurement error. Important questions for future research are to what degree there is agreement among employees within workplaces about the level of change commitment and change efficacy, and whether level of agreement itself may be a predictor of implementation.

Finally, the intervention (HealthLinks), the target practice (workplace health promotion practices), and setting (worksites in low-wage industries in King County, Washington State) may limit the generalizability of the findings. However, there are not theoretical reasons we are aware of that would explain why change commitment and change efficacy would be unrelated to change effort in this context but should be in other contexts.

While our study had limitations, it also had important strengths. We do not know of other studies that have (1) systematically developed and independently validated (including item comprehension, construct validity, scale reliability, and criterion validity) a theory-based measure tailor-made for a specific implementation program and setting; (2) prospectively assessed changes in readiness with measures of program-change effort; and (3) used experimentally manipulated conditions directed at changing readiness factors. We believe this design made for a unique, scientifically rigorous study.

Ultimately, these findings raise more questions than they answer and point to a number of interesting avenues for future research.

## Conclusion

Many implementation theories predict that commitment and efficacy mediate the effect of implementation strategies and actual implementation efforts. We did not find this to be the case in the setting of small worksites in low-wage industries implementing evidence-based health promotion practices. Instead, we found implementation strategies can lead to significant implementation efforts in the absence of improved change commitment—indeed, in the presence of declining change commitment. If replicated—at least in this setting—this suggests that implementation measures and models may be better served by focusing less on attitudinal constructs and more on instrumental constructs, such as planning and technical support.

## Author Contributions

CH, MK, CA, KH, KC, AP, BW, JH, and PH contributed substantially to the conception and design of the study and/or to the acquisition of data. All authors contributed substantially to the analysis and interpretation of findings. CH and PH drafted the article; all authors provided critical revision of the article. All authors provided final approval of the version to be published and all agreed to be accountable for all aspects of the work in ensuring that questions related to the accuracy or integrity of any part of the work are appropriately investigated and resolved.

## Disclaimer

The views expressed in this article are those of the authors and do not necessarily reflect the position or policy of the Department of Veterans Affairs, the National Cancer Institute, or the United States Government.

## Conflict of Interest Statement

The authors declare the research was conducted in the absence of any commercial or financial relationships that could be construed as a potential conflict of interest. The reviewer DH declared a shared affiliation, with no collaboration, with the authors to the handling Editor.
