# Supplementary material for: Readiness to Change Over Time: Change Commitment and Change Efficacy in a Workplace Health-Promotion Trial
Source: Front Public Health. 2018 Apr 23;6:110. doi: 10.3389/fpubh.2018.00110 (PMC5925216; doi:10.3389/fpubh.2018.00110)
Supplement: Supplementary file 1 [file table_1.DOCX]

**Supplementary materials**

Readiness scale and wellness-program effort items

|  | Context (1 = “Strongly Disagree”, 5 = “Strongly Agree”) |
| --- | --- |
|  | 1. The senior leaders are willing to try new things. |
|  | 1. The senior leaders seek ways to improve the work climate. |
|  | 1. The senior leaders reward creativity and innovation in the worksite. |
|  | 1. The senior leaders promote team building to solve worksite problems. |
|  | 1. The managers seek ways to improve the work climate. |
|  | 1. The managers encourage employees to participate in programs. |
|  | 1. Opinion leaders are willing to try new things. |
|  | 1. Opinion leaders seek ways to improve the work climate. |
|  | 1. When we want to try something new we have the training resources to do it. |
|  | 1. When we introduce a new program or change we measure its success by asking employees to fill out a survey about the program. |
|  | **Information Assessment** |
|  | 1. Most employees could take time at work to participate in wellness programs. |
|  | 1. Senior leaders would dedicate financial resources to wellness programs. |
|  | 1. Senior leaders would dedicate staff time to planning wellness programs. |
|  | 1. We have one or more employees who are wellness champions. |
|  | 1. We have one or more senior leaders or managers who are wellness champions. |
|  | **Change Valence** |
|  | 1. Wellness programs would improve employee health in my organization |
|  | 1. Wellness programs reduce employers’ health care costs. |
|  | 1. Wellness programs help companies recruit and retain employees. |
|  | 1. Wellness programs are a good use of financial resources. |
|  | **Change Commitment** |
|  | 1. Our senior leaders are committed to improving our (starting a) wellness program. |
|  | 1. Our opinion leaders are committed to improving our (starting a) wellness program. |
|  | 1. We are motivated to improve our (implement a) wellness program. |
|  | 1. We need to improve our (start a) wellness program within the next year. |
|  | 1. How much time do you think you could spend each week on managing a wellness program? (1. < 30 minutes, 2. 30-59 minutes, 3. 1-2 Hours, 4. 2-3 Hours, 5. > 3 Hours) |
|  | **Change Efficacy** |
|  | 1. We have the skills and expertise to expand our (implement a) wellness program. |
|  | 1. We have enough financial resources to support a wellness program. |
|  | 1. We can (could) manage the politics of implementing a wellness program. |
|  | 1. We can (could) get people to participate in our wellness program. |
|  | **Wellness Program Effort (1 = “No, 5 = “Yes”)** |
|  | 1. Our organization has established, written wellness goals. |
|  | 1. Does [your company] have a worksite wellness committee? |
|  | 1. Does your company have a budget dedicated to health promotion or wellness? |
|  | 1. Does your company have a health promotion or wellness coordinator? |
|  | 1. In an average week, how much time do you spend on managing wellness activities?   (1. < 30 minutes, 2. 30-59 minutes, 3. 1-2 Hours, 4. 2-3 Hours, 5. > 3 Hours) |
